# Design-a-scientist avatar: A new tool for analyzing gender and racial scientist stereotypes

**DOI:** 10.1371/journal.pone.0341309

**Published:** 2026-02-11

**Authors:** Angelina Joy, Channing J. Mathews, Kelly Lynn Mulvey, Adam Hartstone-Rose

**Affiliations:** 1 North Carolina State University, Raleigh, North Carolina, United States of America; 2 The University of Virginia, Charlottesville, Virginia, United States of America; PLOS: Public Library of Science, UNITED KINGDOM OF GREAT BRITAIN AND NORTHERN IRELAND

## Abstract

Representations of scientists have been shown to be influenced by gender and racial/ethnic stereotypes, in which scientists have been typically depicted as White males. Such stereotypes can have negative effects on women and ethnic minoritized individuals’ intention to participate in the science, technology, engineering, and mathematics (STEM) fields. The current study used a new method based on the Draw-a-Scientist task to evaluate US undergraduate students’ (*N* = 371, *M*_age_ = 19.10, *SD* = 1.90) perceptions of scientists via their development of a scientist avatar relative to their explicit STEM stereotypes and color-blind racial attitudes. The skin tone selected for the scientist avatars was also assessed to confirm the utility of the Design-a-Scientist Avatar app. Preliminary results indicated that the saturation and brightness of the skin tones were correlated with scientist avatars described as White or Black. Furthermore, factors such as gender and gender stereotypes were influential for participants’ likelihood of creating a female scientist, and participants’ race/ethnicity, racial/ethnic stereotypes, and color-blind racial attitudes were influential for their likelihood of creating a Black or Latino scientist. The results from this study demonstrate a need for more diverse and inclusive STEM environments and potential interventions to change perceptions around scientists. Moreover, this study introduces a new digital version of the Design-A-Scientist task that can be easily disseminated to collect quantifiable data in the assessment of scientist stereotypes across broader sample populations.

## Introduction

Within the science, technology, engineering, and mathematics (STEM) fields there are gender and racial/ethnic disparities in which women and ethnic minoritized individuals are less likely to pursue STEM degrees and attain STEM careers [1]. Lack of diverse representation within the STEM fields may lead to feelings of less belonging among women and ethnic minoritized people [2,3] and members of these groups may be less likely to see themselves as STEM professionals. Stereotypes about women and ethnic minoritized individuals’ STEM abilities can also deter these groups from participating in STEM. Studies have shown that stereotypical perceptions of STEM professionals, mainly scientists, are present in childhood [4]. However, less is known about young adults’ perceptions of who scientists are and what factors may contribute to more stereotypical perceptions. Thus, the aim of the current study was to assess students’ perceptions of scientists through the creation of a digital version of the classic Draw-a-Scientist task [10]. Further this study tests the utility of this newly developed online avatar design method with a sample of college students as an innovative and easily disseminated method to assess how young adults think about who can be a scientist. Furthermore, our aim was to also explore factors that are related to college students’ perceptions of diverse scientists.

### Factors that predict representations of scientists

In the current study, we examine explicit STEM stereotypes, color-blind racial attitudes [5] and social identities (self-reported gender, race, major) as factors that might explain college students’ representations of scientists.

#### STEM stereotypes.

Stereotypes based on one’s STEM abilities tend to depict women and ethnic minoritized individuals as less competent than men and ethnic majoritized individuals in STEM domains [6–8]. Such stereotypes can negatively impact students’ academic performance. For example, studies on stereotype threat [9,10] have shown that when negative math stereotypes about girls are made salient, female students are more likely to underperform in math. This greater likelihood of experiencing stereotype threat also leads to a greater likelihood of leaving STEM fields for underrepresented groups [11]. Thus, it is important to understand students’ perceptions of STEM professionals and the factors related to these perceptions so scholars and practitioners can expand students’ understandings of who can pursue a STEM career. Furthermore, it is still unknown whether college students’ explicit stereotypes about STEM are related to how they think about who can be a scientist. Thus, this study examines connections between one’s stereotypes about others’ abilities and their perceptions of scientists.

#### Color-blind racial attitudes.

Research has documented the prevalence of color-blind racial attitudes (i.e., unawareness of racial privilege, institutional discrimination and blatant racial issues; Note: The term “color-blind” in this context has ableist connotations, however we use it in the current paper because we used the Color-Blind Racial Attitudes Scale [5] to capture these perceptions) amongst Americans. This work centers on understanding the consequences of a mentality that denies the potential for differences between racial groups, instead focusing on sameness between racial groups [12]. Color-blind thinking is common amongst STEM faculty [13], with findings suggesting that color-blind thinking is associated with STEM instructors not recognizing racialized STEM classroom events [14]. Additionally, college students’ color-blind attitudes are related to their experiences with observing and engaging in racial microaggressions [15]. However, what is not yet known is whether students who hold color-blind racial attitudes are also less likely to design racially diverse scientists. As it is necessary to shift perceptions of who can be a scientist to help increase opportunities for those historically excluded from STEM to enter STEM fields, it may be that color-blind racial attitudes are a key target for intervention to create more inclusive STEM contexts. Thus, if color-blind racial attitudes are related to students’ perceptions of scientists, interventions can be implemented that focus on increasing awareness of issues like institutional discrimination or racial privilege to also promote diverse perceptions of scientists and STEM professionals.

#### Social identities.

We also explore participants’ social identities as potential factors that may be associated with representations of scientists. Prior research with college students [16] has documented that female students were more likely to draw female scientists than were male students, although over 80% of the scientists drawn by female students were still male scientists. Thus, we anticipate that students’ gender identity will be related to their representation of scientist gender. Additionally, prior research with US children has documented that ethnically minoritized children are more likely to use darker skin tones when drawing scientists (Authors), although other work [17] with Colombian and Bolivian students found that even with a sample of students ethnically historically excluded from STEM, almost all students still drew White male scientists. Thus, we are interested in exploring students’ ethnic identities in relation to their representation of scientists. Lastly, we are also interested in exploring whether there are differences in representation based on whether participants are STEM majors or not. Although past research has explored young adults’ perceptions of scientists [16], this study will be the first to do so using a new online expansion of the Draw-a-Scientist task.

### Draw-a-scientist

The Draw-a-Scientist task was developed by David Chambers to assess children’s perceptions of scientists [18]. In this task, children were asked to draw a picture of a scientist on a piece of paper and then the drawing was coded for gender and stereotypical items (i.e., lab coat, science equipment). The findings from this initial study revealed that less than 1% of children drew a female scientist. This study has been replicated over the years and a meta-analysis of 78 studies [4] showed that about 28% of more contemporary children’s (grades k-12) drawings depicted female scientists. However, most of the research using this method only focuses on the gender of the scientist and not the racial attributes included in the scientist depictions. However, another meta-analysis of 30 studies [19] found that 39% of drawings featured a White male scientist, but no information was given about the races of the non-White scientists. It is problematic to analyze the racial attributes depicted in these drawings based simply on the color the child chose to use for the scientist’s skin tone, as children do not have the opportunity to name the race that they have attributed to their drawing. Furthermore, as drawings are typically done on plain white paper, if a participant selects to simply not color the skin of the scientist at all, it may not be appropriate to assume that this reflects representation of a White scientist [20]. Therefore, more research is needed that addresses this gap by also assessing how/if individuals consider race when thinking of scientists in addition to endorsing possible racial/ethnic stereotypes when participants are asked to represent scientists.

In addition to limited considerations of race, the vast majority of prior work with the Draw-A-Scientist task has focused on children and adolescents [4] with only limited prior work with this task focused on older students, for instance emerging adults who are in college. However, the limited prior research that has been conducted has found similar patterns in with emerging adults, with one study [16] sampling over 200 college students documenting that over 80% of drawings represented male scientists, and that female students were more likely to draw female scientists than were male students. Therefore gender stereotypes may still be prevalent into early adulthood, which is a key developmental period when individuals are selecting career pathways [21]. Further, findings suggest emerging adulthood is a key period for persistence in STEM for students historically excluded from STEM fields [22,23].

### Design-a-scientist avatar: a new method for assessing scientist stereotypes

Although the prior work using the Design-A-Scientist task with college students has used the traditional paper-based methodology, it may be important for researchers to adapt this task to better align with emerging adults’ everyday digital experiences. An online, avatar-based version will allow easier dissemination to broader samples of participants, standardization of response options, and direct data capture that will allow both ease and greater objectivity of analysis. To this end, a “Design-a-Scientist Avatar” app was developed for the current study that allows participants to select characteristics for their scientist online (please reach out to the last author for access to this app). In the app, participants are given diverse options for eye color, hair styles, skin tone, facial expression, and articles of clothing and then also directly indicate the gender and race of the scientist they create.

While using paper-based drawings of scientists often makes it hard to interpret the gender or race/ethnicity of the scientist in the picture, with this app we are able to collect both the demographic factors assigned to the scientist avatar by each participant (something not done for race/ethnicity in previous papers) and quantifiable data about selected skin colors (something not easily done in analyses of paper-based methods). While skin tone or color alone is not indicative of race, exploring the skin colors selected provides another avenue to understand how individuals think about who can be scientists. This approach to the color analysis is possible because of the quantifiability of the specific skin color choices: every color can by broken down into its “hue” (the color’s wavelength, or where it falls relative to red, green and blue light), its “saturation” (the color’s intensity from washed out – no saturation – to pure color) and its “brightness” (the relative lightness or darkness of the color; Table 1). Moreover, given the pervasive history of colorism, which centers on bias and discrimination based on the darkness of one’s skin tone [24], new research is needed that moves beyond just exploring explicit racial stereotypes to also explore how individuals may implicitly hold stereotypes about who can be scientists based on skin tone.

**Table 1:** Skin tone colors used in the Design-a-Scientist Avatar app. Modified from a von Luschan scale of 36 colors posted as “Scala cromatica Von Luschan’s, come da modello esposto al Peabody Museum Harvard University No. 2005.1.168” uploaded to Wikimedia Commons by author A7N8X on February 7^th^, 2015. It was downloaded during the creation of the app described in this paper and still posted online as of the time of its submission. Hex code and “RGB” color (i.e., mix of red, green and blue color components) are universally standardized. Hue, saturation and brightness components of the specifically chosen full color options depicted for graphical clarity of how these components combine to form a full color (with an arbitrary magenta [hue = 300] chosen to depict how saturation and brightness affect color composition).

von Luschan Number	Hex Code	RGB	Hue^a^		Saturation^b^		Brightness^c^		Full Color Option
1	f1eacd	241,234,205	48		15		95		
3	eadacb	234,218,203	29		13		92		
4	eec479	238,196,121	38		49		93		
8	e7c594	231,197,148	35		36		91		
12	e79e79	231,158,121	20		48		91		
16	db8f46	219,143,70	29		68		86		
20	e8af68	232,175,104	33		55		91		
23	d37636	211,118,54	24		74		83		
27	8f4735	143,71,53	12		63		56		
30	883b34	136,59,52	5		62		52		
34	503a39	80,58,57	3		29		31		
36	272b30	39,43,48	213		19		19		

^a^On the 360-degrees color wheel. Saturation and brightness at 100%.

^b^Out of 100%. Hue arbitrarily at 300 and brightness at 100%.

^c^Out of 100%. Hue arbitrarily at 300 and saturation at 100%.

These quantifiable axes of skin tone selected for the scientist avatars will be assessed to see if there are associations between the skin tones participants choose and the explicit race/ethnicity they ascribe for their scientist, allowing us to attempt to validate the relationship between ascribed race/ethnicity of the scientists and skin color assigned to them by using these more defined attribute options, thus avoiding many of the assumptions and subjective scoring inherent in the foundational Draw-a-Scientist research. If validated, this adaptation of the Draw-A-Scientist tasks will allow scholars to better understand how young adults today view scientists, including assessing how both race and gender stereotypes about scientists predict the representation of scientists from traditionally excluded backgrounds.

### Current study

The present study aims to evaluate college students’ gender and racial/ethnic stereotypes about scientists as well as examine which factors, explicit STEM stereotypes and racial attitudes, are associated with the gender and race of the scientist avatar they created. This study specifically recruited college students as many students drop out of STEM majors during college, especially underrepresented students [25]. Additionally, past research has examined college students’ gendered perceptions of scientists [16] but not their perceptions of scientists’ race/ethnicity. Therefore, to create interventions to increase student retention in STEM in college it is important to first understand if they have stereotypical racial/ethnic perceptions of scientists. Furthermore, this study uses the Design-a-Scientist Avatar app, a new iteration of the Draw-a-Scientist task, in which participants create a scientist avatar online. To confirm the utility of this tool, associations between the skin tone selected for the scientist avatars and the ascribed race/ethnicity of the scientist avatars will be assessed. Therefore, this app could be a new tool to objectively capture people’s perceptions of scientists and confirm congruence of the designed and directly reported race/ethnicity and gender attributes.

### Hypotheses

1) Past research with college students has shown that girls are more likely to represent female scientists [16], thus it is hypothesized that participants will be more likely to design scientist avatars that match their own gender and racial/ethnic identity.
2) The more likely participants are to believe that a particular group (girls, boys, historically excluded racial/ethnic groups (i.e., Black and Latino), historically well represented racial/ethnic groups (i.e., White and Asian) will do well in STEM, the more likely they will be to design a scientist that reflects those groups.
3) Participants who hold color-blind racial attitudes (i.e., are less aware of racial privilege, institutional discrimination, and blatant racial issues) will be less likely to create a Black, or Latino scientist.
4) The reported race/ethnicity of the scientist avatars would be correlated to the hue, brightness, and saturation of the skin tone selected.

Additionally, because there are still racial/ethnic disparities within STEM fields [1], we also explore whether differences in representations of scientist emerge between STEM majors and those who are not STEM majors, although although this is an open question and we do not have a firm hypothesis. Finally, we controlled for participant age.

## Method

### Participants

Participants were college students from a large predominantly White public university in the Southeastern United States (*N* = 371, *M*_age_ = 19.10, *SD* = 1.90). 55.2% of participants identified as female, 35.5% identified as male, 8% did not report their gender, and 1.2% identified as other. Only participants who identified as female or male were included in this study. Of the participants that chose “other” as their gender half of them created a scientist with a gender as “other.” We acknowledge that the use of “other” as a gender category should not be used in future studies as this term may marginalize those from trans and gender-nonconforming communities. Furthermore, future work should continue to explore representation of non-binary scientists and how non-binary individuals think about who can be scientists. 67.4% of participants self-identified as White/European American, 10.1% as Asian/Asian American, 4.2% as Black/ African American, 5.0% as Hispanic/Latino, 3.4% as Bi-racial/Multi-racial, 1.6% as “other”, 0.8% as American Indian/Native American, and 7.4% did not report their race/ethnicity. 41.6% of participants had a STEM major.

### Procedure

This study was approved by the IRB at North Carolina State University (22449). Written consent was obtained. Participants were recruited as part of introductory Psychology classes (which serve students across a wide range of majors). Participants were recruited from March 19, 2021 to December 7, 2021 and participants gave written consent through the Qualtrics survey. Participants first completed the Design-a-Scientist Avatar task then a Qualtrics survey online and received class credit for participating in the study. Of the 544 participants that completed the Qualtrics survey, our analysis was limited to the 371 who also completed the Design-a-Scientist Avatar task. Files are available on OSF. https://doi.org/10.17605/OSF.IO/UH52S.

### Measures

#### Design-a-scientist avatar.

***Scientist skin tone*.** Participants were instructed to create a scientist on the Design-a-Scientist Avatar app. On the app participants were given 12 different skin tone colors to choose from (see Table 1). The skin tones were based on the von Luschan’s Skin Color Scale [26] – a scale developed at the end of the nineteenth century in an effort to create a standardized way to record human skin colors. This approach relied on tiles numbered roughly from light to dark (e.g., along the brightness axis) but with seemingly random a) intervals between consecutive colors and b) variation in tone (see Table 1). While this effort to standardize measurement of skin tone led to some improvement of consistency and reproducibility, the colored tiles were not able to be consistently reproduced and this approach was abandoned in favor of more consistent methods based on reflectance spectrophotometry [27]. However, versions of the von Luschan scale are still relatively pervasive (e.g., as online results of searches for “skin tone scale”) and contain more variation (especially in the hue axis) than other scales (e.g., the Fitzpatrick and Monk scales). While many of the original tile versions have, over the more than a century since their production, become apparently faded (as are some of the digitized versions likely based on them), we selected 12 of the 36 von Luschan colors (roughly every third) from one of the versions posted on Wikimedia Commons (Image posted as “Scala cromatica Von Luschan’s, come da modello esposto al Peabody Museum Harvard University No. 2005.1.168” uploaded by author A7N8X on February 7th, 2015. Downloaded during the creation of the app and still posted online as of the time of submission) to serve as the color options in the app. These were shown to participants in a scrambled order, that is, not in sequence along any of the hue, saturation and brightness quantified axes, though their selections were ultimately analyzed according to these axes.

As noted above, all colors can be described according to several standard and reproducible ways (Table 1). For instance, in digital applications, colors are generally signified by their “hex code”, a combination of six alphanumeric combinations –with each of the three pairs signifies the red, green and blue color component coded according to the hexadecimal system. An additional approach to categorize colors is more directly according to their red, green and blue components, or so called “RGB” values from 0 to 255 – a scale that reflects their common storage as 8-bit values.

While hex codes and RGB values allow for easy reproducibility of specific colors (e.g., guaranteeing that the colors reproduced in the digital version of this paper, if coded correctly, should be accurate across platforms) the hue, saturation and brightness (or so called “HSB” values) are more pertinent to this study: although most people have skin colors from a fairly narrow band of hues (i.e., most are between the red and yellow ranges) there is great variability in the saturation and brightness of human skin tones. That is, people’s pigmentation ranges from extremely pale (i.e., less brightness and saturation) to very dark (i.e., more brightness and saturation). As past research has not examined racial/ethnic stereotypes using the Draw-a-Scientist task, the Design-A-Scientist Avatar task is a first step in assessing racial/ethnic stereotypes using a more nuanced approach.

***Scientist race/ethnicity*.** After they completed their scientist avatar, participants were asked to select the race/ethnicity of their scientist avatar from the following eight choices: Black/African American, White/European American, Hispanic/Latino, American Indian/ Native American, Asian/Asian American, Bi-racial/Multi-racial, Pacific Islander, or Other. For the purposes of these analyses only scientist avatars identified as Asian, Black, Latino, or White were used, the rest were omitted as very few or no students used these categories (*N* excluded = 40).

***Scientist gender*.** Participants were given three choices (Female, Male, Other) and were asked to select a gender for their scientist avatar. For the purposes of these analyses, avatars of scientists that were not identified as female or male were omitted (*N* excluded = 6).

#### STEM stereotypes-race/ethnicity.

Participants rated how much they agreed with 4 items [28] capturing the performance of 4 different racial/ethnic groups in STEM (Asian, Black, White, Latino) on a scale of 0–100. An example item is: “I think that Asian/Black/Latino/White students should do well in STEM.”

#### STEM stereotypes-gender.

Participants rated how much they agreed with 2 items [28,29], one capturing boys’ performance in STEM and one capturing girls’ performance in STEM (example item: “I think that girls/boys should do well in STEM”), on a scale of 0–100.

#### Color-blind racial attitudes.

Participants completed 3 subscales of the color-blind racial attitudes scale [5]:

1. **Unawareness of racial privilege:** rated 7 items such as “Everyone who works hard, no matter what race they are, has an equal chance to become rich” on a scale of 1 (strongly disagree) to 6 (strongly agree; *a* = 0.87).
2. **Unawareness of institutional discrimination:** rated 7 items such as “Social policies, such as affirmative action, discriminate unfairly against White people” on a scale of 1 (strongly disagree) to 6 (strongly agree; *a* = 0.83).
3. **Unawareness of blatant racial issues:** rated 6 items such as “Racism may have been a problem in the past, but it is not an important problem today” on a scale of 1 (strongly disagree) to 6 (strongly agree; *a* = 0.83).

### Data analyses

To examine participants’ choices in skin tone and race/ethnicity for their scientist, correlations between skin tone hue, saturation, brightness and the race/ethnicity of the scientists were computed.

Descriptive statistics and correlations were first computed, then preliminary ANOVAs were used to examine any gender or racial/ethnic differences in the Color-Blind Racial Attitudes Scale. A MANOVA was used to examine any gender or racial/ethnic differences in the STEM Stereotype measures. Aligned with our hypotheses, only two-way interactions (race, gender, STEM major) were examined. For the purposes of these analyses only the Asian/Asian American, Black/African American, Hispanic/Latino, and White/European American racial/ethnic groups were used. Then a logistic regression was used to determine what factors (age, gender, race, STEM major, Female STEM Stereotype Endorsement, Male STEM Stereotype Endorsement) were related to the assigned gender of the scientist avatar. Finally, a multinomial logistic regression was used to determine what factors (age, gender, race, STEM major, Asian students STEM Stereotype Endorsement, Black students STEM Stereotype Endorsement, Latino students STEM stereotype Endorsement, White students STEM Stereotype Endorsement, Unawareness of Racial Privilege, Unawareness of Institutional Discrimination, Unawareness of Blatant Racial Issues) were related to creating an Asian scientist, a Black or Latino scientist, or a White scientist. A power analysis for regressions was conducted using G*Power anticipating an effect size of 0.15, with the alpha at 0.05, power of 0.80, and number of predictors set to 14. It was determined that a sample size of at least 55 was required. While we recognize that this grouping is a limitation of the current work, as we did not have enough Black and Latino representation to analyze them independently, we grouped Black and Latino scientists together given their historical exclusion from STEM.

## Results

### Descriptives

#### Correlations.

STEM stereotypes about boys and girls were positively correlated with each other as well as with the four racial STEM stereotypes. All four racial STEM stereotypes were positively correlated with each other, and the three color-blind racial attitudes measures were also positively correlated with each other (see Table 2).

**Table 2:** Correlations.

	1	2	3	4	5	6	7	8	9
1. STEM Stereotypes Girls	–								
2. STEM Stereotypes Boys	0.83**	–							
3. STEM Stereotypes Asian Students	0.84**	0.84**	–						
4. STEM Stereotypes Black Students	0.88**	0.86**	0.93**	–					
5. STEM Stereotypes Latino Students	0.86**	0.85**	0.91**	0.96**	–				
6. STEM Stereotypes White Students	0.87**	0.85**	0.94**	0.96**	0.93**	–			
7. Unawareness of Racial Privilege	−0.04	0.05	0.03	−0.01	−0.01	0.01	–		
8. Unawareness of Institutional Discrimination	−0.01	0.08	0.09	0.03	−0.01	0.05	0.67**	–	
9. Unawareness of Blatant Racial Issues	−0.05	0.06	0.07 = 6	−0.01	−0.01	0.01	0.76**	0.78**	–

#### STEM stereotype means.

***Gender STEM stereotypes***. Based on results from a MANOVA there were no differences between participants’ ratings of STEM competence for boys and girls (*F*(1, 276) = 1.31, *p* = 0.25, partial η^2^ = 0.01). There were also no mean differences based on the gender *(F*(1, 276) = 0.86, *p* = 0.36, partial η^2^ = 0.003), race/ethnicity (*F*(3, 276) = 0.14, *p* = 0.94, partial η^2^= 0.002), or STEM major (*F*(1, 276) = 0.34, *p* = 0.56, partial η^2^= 0.001) of the participants (See Table 3 for overall means, standard deviations and ranges). None of the interactions were significant.

**Table 3:** Means, Standard Deviations, and Ranges.

	M	SD	Range
STEM Stereotypes Girls	72.04	25.35	0-100
STEM Stereotypes Boys	68.97	26.54	0-100
STEM Stereotypes Asian Students	74.14	25.66	0-100
STEM Stereotypes Black Students	71.41	25.59	0-100
STEM Stereotypes Latino Students	70.48	25.37	0-100
STEM Stereotypes White Students	71.73	25.44	0-100
Unawareness of Racial Privilege	2.99	1.19	1-6
Unawareness of Institutional Discrimination	2.63	1.01	1-6
Unawareness of Blatant Racial Issues	2.20	1.04	1-6

***Racial/ethnic STEM stereotypes***. The results from the MANOVA indicated that there were no mean differences between participants’ ratings of STEM abilities of Asian, Black, Latino, or White students (*F*(3, 819) = 0.21, *p* = 0.89, partial η^2^ = 0.001). There were also not any differences based on the participants’ gender (*F*(3, 819) = 1.66, *p* = 0.18, partial η^2^ = 0.01) or based on whether participants were a STEM major or not (*F*(3, 819) = 0.96, *p* = 0.41, partial η^2^= 0.003) and the interactions were also not significant. However, there were mean differences based on participants’ race/ethnicity (*F*(9, 819) = 3.02, *p* = 0.001, partial η^2^ = 0.03).

Asian participants rated Asian students (*M* = 72.93, SD = 25.26, *p* < 0.001) higher than Black (*M* = 65.37, SD = 24.72), Latino (*M* = 63.41, SD = 22.06), and White students (*M* = 66.48, SD = 24.52). White participants rated Asian students (*M* = 74.88, SD = 25.90) higher than Black (*M* = 72.95, SD = 25.71, *p* = 0.01), Latino (*M* = 72.02, SD = 25.78, *p* < 0.001), and White students (*M* = 73.36, SD = 25.47, *p* = 0.01). Hispanic/Latino participants rated Latino students (*M* = 59.02, SD = 27.14) lower than Asian students (*M* = 66.98, SD = 31.82, *p* = 0.03) and White students (*M* = 66.20, SD = 30.91, *p* = 0.02), but did not differ in their ratings of Black students (M = 62.73, SD = 28.82). There were no differences in Black participants’ ratings for students from any racial group (See Table 3 for overall means, standard deviations and ranges).

#### Color-blind racial attitudes means.

***Unawareness of racial privilege*.** there were gender (*F*(1, 287) = 13.06, *p* < 0.001, partial η^2^ = 0.04) and racial/ethnic differences (*F*(3, 287) = 3.85, *p* = 0.01, partial η^2^ = 0.04) in participants’ unawareness of racial privilege. However, there were not STEM major differences (*F*(1, 287) = 0.32, *p* = 0.57, partial η^2^ = 0.001) or significant interactions. Men (*M* = 3.46, SD = 1.14) had less awareness of racial privilege compared to women (*M* = 2.72, SD = 1.08, *p* < 0.001). Black participants (*M* = 2.31, SD = 0.85) had more awareness of racial privilege compared to White participants (*M* = 3.10, SD = 1.19, *p* = 0.01). There were no differences for Hispanic/Latino participants (*M* = 2.62, SD = 1.33) or Asian participants (*M* = 2.94, SD = 0.82; See Table 3 for overall means, standard deviations and ranges).

***Unawareness of institutional discrimination***. There were gender (*F*(1, 285) = 15.32, *p* < 0.001, partial η^2^ = 0.05) and racial/ethnic differences (*F*(3, 285) = 3.45, *p* = 0.02, partial η^2^ = 0.04) in awareness of institutional discrimination. However there were not any differences based on STEM major (*F*(1, 285) = 0.47, *p* = 0.49, partial η^2^ = 0.002) and there were not significant interactions. Men (*M* = 3.16, SD = 0.94) had less awareness of institutional discrimination compared to women (*M* = 2.31, SD = 0.89, *p* < 0.001). Black participants (*M* = 2.12, SD = 0.70) had more awareness of institutional discrimination than Asian participants (*M* = 2.84, SD = 0.95 *p* = 0.05) or White participants (*M* = 2.69, SD = 1.02, p = 0.02). There were no differences for Hispanic/Latino participants (*M* = 2.24, SD = 0.99; See Table 3 for overall means, standard deviations and ranges).

***Unawareness of blatant racial issues*.** There were gender (*F*(1, 288) =14.39, *p* < 0.001, partial η^2^ = 0.05) and racial/ethnic differences (*F*(2, 288) =2.95, *p* = 0.03, partial η^2^ = 0.03) in awareness of blatant racial issues. However, there were not differences based on STEM major (*F*(1, 288) = 0.48, *p* = 0.49, partial η^2^ = 0.002) and there were not significant interactions. Men (*M* = 2.68, SD = 1.02) had less awareness of blatant racial issues compared to women (*M* = 1.91, SD = 0.92, *p* < 0.001). Black participants (*M* = 1.62, SD = 0.63) had more awareness of blatant racial issues compared to White participants (*M* = 2.26, SD = 1.04, *p* = 0.02). There were no differences for Hispanic/Latino (*M* = 1.80, SD = 1.11) or Asian participants (*M* = 2.33, SD = 0.96; See Table 3 for overall means, standard deviations and ranges).

#### Scientist avatars.

41.2% of participants created a scientist they described as male and 58.8% created a scientist they described as female. 70.3% of participants described their scientist as White, 16.5% as Black, 9.4% as Asian, and 3.9% as Latino (see Table 4 for full results). Furthermore, providing support for Hypothesis 1, 70.5% of self-described Asian participants described their scientist avatar as Asian, 86.7% of Black participants created a Black scientist, 38.8% of Latino participants created a Latino scientist, and 83.5% of White participants created a White scientist. 83.5% of male participants created a male scientist and 86.4% of female participants created a female scientist.

**Table 4:** Percent of Scientist Avatars.

	Percent of Avatars (Female Participants (N = 206))	Percent of Avatars (Male Participants (N = 133)	Percent of Avatars (Asian Participants (N = 29))	Percent of Avatars (Black Participants (N = 51))	Percent of Avatars (Latino Participants (N = 12))	Percent of Avatars (White Participants (N = 216))	Percent of Avatars (Total = 371)
**Scientist Avatar Gender**							
Female Scientist	86.4	16.5	50.0	40.0	61.1	59.2	58.8
Male Scientist	13.6	83.5	50.0	60.0	38.9	40.8	41.2
**Scientist Avatar Race/Ethnicity**							
Asian	8.1	11.4	70.5	0.0	0.0	2.2	9.4
Black	17.8	14.6	8.9	86.7	16.7	12.6	16.5
Latino	4.9	2.4	2.9	0.0	38.8	1.7	3.9
White	69.2	71.5	17.7	13.3	44.5	83.5	70.3

#### Scientist avatars’ skin tone.

To test Hypothesis 4, correlations between description of racial group and skin tone selected were computed. Scientist avatars with brighter and less saturated skin tones were more likely to be described as White. Scientist avatars with more saturated skin tones were more likely to be described as Black or Asian. Additionally, scientist avatars with less bright skin tones were more likely to be described as Black (see Table 5). As participants that described their avatars as Latino selected more diverse skin tone options, this race/ethnicity descriptor did not correlate to any of the numerical color descriptors. Furthermore, there was no correlation between any race/ethnicity and selections along the hue spectrum.

**Table 5:** Scientist Avatars Race/Ethnicity Skin Tone Correlations.

Scientists Explicit Race/Ethnicity	Hue	Saturation	Brightness
Asian Scientist	−0.08	0.19**	0.003
Black Scientist	−0.03	0.44**	−0.77**
Latino Scientist	−0.05	0.10	0.06
White Scientist	0.09	−0.53**	0.59**

#### Scientist avatars’ gender.

Testing Hypothesis 1 and 2, we computed a logistic regression to identify the odds of creating a female scientist by STEM major (yes, no), participant gender and participant STEM stereotypes, controlling for participant age. Findings from the logistic regression revealed that the odds of creating a female scientist were higher if the participants were female (AOR = 42.73, *p* < 0.001) and if participants had higher endorsement that girls should do well in STEM (AOR = 1.03, *p* = 0.03). However, the odds of creating a female scientist were lower if participants had higher endorsement that boys should do well in STEM (AOR = 0.97, *p* = 0.03; see Table 6).

**Table 6:** Logistic Regression for the Odds of Creating a Female Scientist Avatar as Compared to a Male Scientist Avatar.

	B	SE	Wald	df	Sig	Exp(b)
Age	0.01	0.11	0.01	1	0.91	1.01
Gender	3.76	0.36	107.40	1	0.001	42.73
Asian	−0.96	0.93	1.05	1	0.31	0.38
Black	−0.53	1.10	0.22	1	0.64	0.59
Latino	−1.26	1.10	1.31	1	0.25	0.28
White	−1.10	0.83	1.74	1	0.19	0.33
STEM Major	0.14	0.35	0.17	1	0.68	1.15
STEM Stereotypes Girls	0.03	0.01	4.49	1	0.03	1.03
STEM Stereotypes Boys	−0.03	0.01	4.54	1	0.03	0.97

Note: Participant Age = ages in years, Participant Gender: 0 = male, 1 = female.

#### Scientist avatars’ race/ethnicity.

Testing Hypothesis 1, 2 and 3, we computed logistic regression analyses predicting the odds that participants would create a scientist of a particular race based on their scores on their demographics (gender, race and STEM major (yes, no)), their STEM racial stereotypes, and their COBRAs scores, controlling for participant age, Models were computed with each racial group as the reference group. Participants who rated Asian students’ abilities in STEM higher were more likely to create an Asian scientist as compared to a White scientist (AOR = 0.92, *p* = 0.01, see Table 7). Participants who showed greater unawareness of institutional discrimination were less likely to create a Black or Latino scientist (AOR = 0.40, *p* = 0.001) as compared to a White scientist. Participants who were Black were more likely to create a Black or Latino scientist as compared to a White scientist (AOR = 0.01, *p* < 0.001; see Table 8). Including participant race in the model assessing comparisons between creating Black or Latino scientists and Asian scientists was leading to unusual high adjusted odds ratios, likely due to the fact that almost no Asian participants created an Avatar of a Black or Latino scientist. Thus, for this comparison, we removed participant race from the model, see Table 9. This analysis revealed that participants were more likely to create a Black or Latino scientist as compared to an Asian scientist if they thought that Latino scientists should be good at STEM (AOR = 1.09, *p* = .014) and were less likely to create a Black or Latino scientist as compared to an Asian scientist if they thought Asian scientists should be good at STEM (AOR = 0.911, *p* = .003). Finally, participants were less likely to create a Black or Latino scientist as compared to an Asian scientist if they were more unaware of institutional discrimination (AOR = .35, *p* = .026).

**Table 7:** Logistic Regression for the Odds of Creating an Asian Scientist Avatar as Compared to Creating a White Scientist Avatar.

	B	SE	Wald	df	Sig	Exp(b)
Age	−0.08	0.21	0.16	1	0.69	0.92
Gender	0.54	0.64	0.71	1	0.40	1.72
Asian	−19.09	1464.11	0.00	1	0.99	<0.001
Black	−0.75	0.00	1	1	–	0.47
Latino	0.18	2108.07	0.00	1	1.00	1.20
White	−13.48	1464.11	0.00	1	0.99	<0.001
STEM Major	0.53	0.59	0.80	1	0.37	1.69
STEM Stereotypes Asian Students	0.08	0.03	7.75	1	0.01	1.09
STEM Stereotypes Black Students	−0.05	0.05	1.26	1	0.26	0.95
STEM Stereotypes Latino Students	−0.03	0.04	0.70	1	0.40	0.97
STEM Stereotypes White Students	−0.02	0.04	0.27	1	0.60	0.98
Unawareness of Racial Privilege	0.05	0.38	0.02	1	0.90	1.05
Unawareness of Institutional Discrimination	−0.74	0.44	2.88	1	0.09	0.48
Unawareness of Blatant Racial Issues	−0.15	0.52	0.09	1	0.78	0.86

Note: Participant Age = ages in years, Participant Gender: 0 = male, 1 = female.

**Table 8:** Logistic Regression for the Odds of Creating a Black or Latino Scientist Avatar as Compared to Creating a White Scientist Avatar.

	B	SE	Wald	df	Sig	Exp(b)
Age	−0.01	0.11	0.01	1	0.92	0.99
Gender	−0.04	0.38	0.01	1	0.91	0.96
Asian	−1.36	0.93	2.17	1	0.14	0.26
Black	−4.72	1.28	13.67	1	<0.001	0.01
Latino	−1.50	0.81	3.46	1	0.06	0.22
White	0.46	0.71	0.41	1	0.52	1.58
STEM Major	0.20	0.32	0.39	1	0.53	1.22
STEM Stereotypes Asian Students	0.03	0.02	1.09	1	0.15	1.03
STEM Stereotypes Black Students	−0.03	0.03	1.20	1	0.27	0.97
STEM Stereotypes Latino Students	0.01	0.02	0.42	1	0.52	1.01
STEM Stereotypes White Students	−0.02	0.02	1.21	1	0.27	0.98
Unawareness of Racial Privilege	0.14	0.21	0.40	1	0.53	1.14
Unawareness of Institutional Discrimination	−0.91	0.28	10.39	1	0.001	0.40
Unawareness of Blatant Racial Issues	0.34	0.30	1.39	1	0.24	1.41

Note: Participant Age = ages in years, Participant Gender: 0 = male, 1 = female.

**Table 9:** Logistic Regression for the Odds of Creating a Black or Latino Scientist Avatar as Compared to Creating an Asian Scientist Avatar.

	B	SE	Wald	df	Sig	Exp(b)
Age	−.09	0.19	0.25	1	0.62	0.91
Gender	−0.52	0.64	0.65	1	0.42	0.60
STEM Major	−.797	0.55	2.09	1	0.15	2.22
STEM Stereotypes Asian Students	−0.09	0.03	8.90	1	0.003	0.91
STEM Stereotypes Black Students	−.001	0.04	0.001	1	0.98	0.99
STEM Stereotypes Latino Students	0.09	0.04	6.05	1	0.014	1.09
STEM Stereotypes White Students	0.01	0.04	0.001	1	0.98	0.99
Unawareness of Racial Privilege	0.65	0.40	2.63	1	0.10	1.91
Unawareness of Institutional Discrimination	−1.05	0.47	4.99	1	0.26	0.35
Unawareness of Blatant Racial Issues	0.32	0.51	0.40	1	0.53	1.38

Note: Participant Age = ages in years, Participant Gender: 0 = male, 1 = female.

## Discussion

This study provides new information on young adults’ perceptions of scientists and was a pilot validation to assess the adequacy of a new Design-a Scientist Avatar app. Results indicated that there were similarities in the skin tones being chosen for scientists described as Black and in the skin tones for scientists described as White. Therefore, this is promising preliminary evidence that this new online task can be used to assess scientist racial/ethnic stereotypes, although we would recommend that scholars use both the app and an explicit question about race/ethnicity of the scientist as further validation studies are conducted. Furthermore, explicit STEM stereotypes were important factors for whether participants created a female or a male scientist, and color-blind racial attitudes played a role in whether young adults designed a White, Asian, Black, or Latino scientist. Thus, this study provides both new insight into young adults’ perceptions of scientists as well as a preliminary validation of a new method that could be used in future research to continue collecting children’s and adults’ representations of scientists.

### STEM stereotypes

Our findings revealed that there were no differences in how participants rated girls’ and boys’ abilities in STEM on explicit measures, although they demonstrated ingroup preference in designing scientists, with male participants predominantly representing male scientists and female participants predominantly representing female scientists. Moreover, STEM stereotypes did predict if a participants designed a male or female scientist, with those holding higher perceptions that girls should be good at STEM more likely to draw a female scientist and those holding higher perceptions that boys should be good at STEM less likely to draw a female scientist, see additional discussion below.

Additionally, there were racial/ethnic differences in terms of the racial/ethnic STEM stereotypes: participants generally rated Asian students higher in their STEM abilities in comparison to the other racial/ethnic groups. This finding is likely related to the model minority myth, in which Asian students are expected to naturally excel in STEM [30]. However, research has also shown that there are differences between Asian subgroups, for example, in academic performance and selection of STEM majors [31]. Although the current study did not have enough Asian participants to conduct ethnic-subgroup analyses, future research should examine these differences more indepth to have a more nuanced understanding of perceptions of different Asian student groups in STEM.

These results indicate that young adults may hold more racial/ethnic stereotypes about STEM than gender stereotypes about STEM. These results also reflect current statistics showing that about 50% of STEM degrees today are being earned by women, however less than 20% of STEM degrees are being earned by Black or Latino students [1]. Thus, our participants’ gender stereotype beliefs may be reflective of a more gender inclusive STEM environment, while their racial/ethnic stereotypes may also be reflective of a less racial/ethnic diverse environment. Furthermore, participants’ more equitable gender stereotype beliefs may also be reflective of researchers’ and schools’ increased efforts to change perceptions of girls’ abilities in STEM and keep girls engaged in STEM throughout school, however more efforts may be needed to create equitable perceptions based on race/ethnicity. Interventions aiming to decrease traditional STEM stereotypes may need to spend more time on racial/ethnic stereotypes and demonstrate that people of all racial/ethnic backgrounds are capable of doing well in STEM.

### Color-blind racial attitudes

Previous research has mixed findings in regards to gender differences in color-blind racial attitudes, with some research showing no differences in college students’ attitudes [32] and others finding that college aged White men tend to have greater color-blind racial attitudes than college aged White women [33]. Our results with a predominantly White sample demonstrated that, in general, men tended to have less awareness of racial privilege, institutional discrimination, and blatant racial issues in comparison to women.

We also found that White participants had less awareness of racial privilege, institutional discrimination, and blatant racial issues compared to Black participants. Asian participants tended to have less awareness of institutional discrimination in comparison to Black participants. This result is in line with previous work which has found that White participants show less awareness of racial privilege, institutional discrimination, and blatant racial issues compared to participants of color [32]. As for Asian participants, the model minority myth may play a role in this finding [30,34]. Asian students who internalize the model minority myth may be more likely to endorse color-blind racial attitudes as a way to uphold the belief in success through individual efforts rather than acknowledging the role of structural racism [34]. However, research has showed that students who participated in diversity courses and activities showed a greater decrease in their color-blind racial attitudes over their four years at the university than those who did not participate in diversity activities [33]. Additionally, close friendships with Black peers were also related to decreases in color-blind racial attitudes over time [33]. Therefore, intergroup contact and diversity trainings may be important avenues for increasing awareness of racial privilege, institutional discrimination, and blatant racial issues among male college students as well as White and Asian students.

### Scientist avatars’ skin tone

There was no correlation between the races/ethnicities participants ascribed to their scientist avatars and the hue of the color (i.e., the component ranging from yellows, oranges and reds) of the skin tone chosen for their avatars. In part, this is probably influenced by the fact that most of the von Luschan colors exhibit a fairly narrow hue range, but it also may relate to the stronger influence of the amount and depth of color – biologically speaking, the amount of melanin present in the skin. Thus, it is not surprising that while hue did not correlate to race/ethnic descriptors, there were significant signals in the saturation and brightness color components.

In general, participants were more likely to use more saturated skin tones for scientist avatars described as Asian and Black and less saturated skin tones for avatars described as White. Additionally, brighter skin tones were positively associated with White scientist avatars and negatively associated with Black scientist avatars. Therefore, saturation and brightness values may be ways in which we can quantitatively examine the relationship between selected skin tones and represented scientist race/ethnicity in studies using digital measures of identity – especially for those who are representing Black/African American and While/European American scientists. However, no factors of skin tone were related to scientist avatars described as Latino/Hispanic and the correlations were weak (low for saturation and not significant for brightness) for avatars described as Asian/Asian American. While this may be because of the limited number of participants who indicated that they represented Latino scientists, it is also likely that the reduced relationship between color variables and ascribed race/ethnicity in these groups is because of the greater variability in skin tone colors for these groups, and Latinos in particular [35]. Thus it is critically important to assess both the skin tone and the explicit racial categories participants identify for their scientist in order to more accurately capture how individuals assign race as a social identity across varied skin tones. Future research should use this tool with larger samples of Hispanic/Latino and Asian/Asian American populations, perhaps with greater specificity of geographic ancestry, to better capture perceptions of scientists from these backgrounds. Additionally, future research might explicitly ask participants to design a scientist who is Hispanic/Latino or who is Asian/Asian American to explore these questions with a greater sample of representations of scientists drawn to be Hispanic/Latino and Asian/ Asian American. Finally, future research might use this tool to more carefully detangle stereotypes based on skin tone and stereotypes based on race/ethnicity, aligned with the findings suggesting that colorism is the root of much discrimination [24], but is likely underexamined within STEM contexts.

The results from this study demonstrate that there may be merit in this new Design-a-Scientist Avatar app. By demonstrating that there are similarities in how participants are creating scientists based on race/ethnicity we are better able to understand the perceptions people have about scientists. Furthermore, this preliminary evidence suggests that the app provides more accurate and precise information than the original paper-based task, that can be used to help analyze stereotypes related to scientists in more quantifiable ways. Thus, after additional validation this app can be more widely used in research. For example, the app can be used to assess stereotypes of broadly defined scientists or more specific perceptions, such as those of engineers or computer scientists which typically have less gender and racial/ethnic diversity in comparison to the other STEM fields [1].

### Scientist avatars’ gender

We found that women were more likely than men to create a female scientist when creating their scientist avatar. This supports previous findings that show girls from all age groups are more likely than boys to draw a female scientist [4,16]. Additionally, men were more likely to create male scientists, which supports our hypothesis of ingroup preference playing a role in gender representations of scientists. However, the more participants endorsed beliefs that girls should do well in STEM, the more likely they were to create a female scientist. On the other hand, participants who tended to endorse beliefs that boys should do well in STEM were more likely to create a male scientist. Thus, more than just ingroup preferences is at work in thinking about representing scientists: one’s beliefs about other’s abilities based on their gender may also contribute to their own perceptions of who can participate in science.

### Scientist avatars’ race/ethnicity

Lastly, we assessed the race of the scientist avatars. In general, ingroup preferences were common amongst participants, particularly with Black and Latino participants being more likely than White participants to create a Black or Latino scientist. However, Latino participants had the lowest percentage (38.8%) of avatars with the same race/ethnicity as themselves. Perhaps efforts to increase diverse representations of scientists have lacked Latino representation, therefore Latino participants may not see themselves as scientists to the same degree as Black participants. In fact, after a group of Hispanic middle school students participated in a Space Science Education Program researchers found that the students had an increase in positive attitudes toward science, however there was no significant increase in the desires to become scientists and only 12.8% of students believed that scientists were “like people they knew” [36]. Sorge, Newsom [36] believed these results may be due to negative stereotypes as well as a lack of Hispanic role models in science-related fields. Furthermore, research with Latino high school students found that students who participated in a program with both role model and self-affirmation interventions were more likely to believe that people from their ethnic background were likely to become scientists [37]. Therefore, to improve Latino students’ perceptions of STEM, efforts may need to highlight Latino representation while also increase students’ STEM-identity.

Additionally, participants who had less awareness of institutional discrimination were less likely to create a Black or Latino scientist compared to a White scientist. Being more aware of institutional discrimination may allow students to understand the racial/ethnic disparities within the STEM fields and may promote more inclusive perceptions of who participates in STEM. Moreover, aligning with previous work [28], Black participants had more awareness of institutional discrimination in comparison to White and Asian participants. This finding may also suggest a need for interventions to also address color-blind attitudes, especially among White and Asian students as they have been historically well-represented in STEM.

Also, participants who endorsed beliefs that Asian students should do well in STEM were more likely to draw an Asian scientist compared to a White scientist. Therefore, endorsing traditional stereotypes that depict Asian students as having higher abilities in STEM is related to also perceiving scientists as Asian. According to National Science Board [38] Asians are also overrepresented in STEM with Asians comprising of 6% of the U.S. workforce, but 9% of the STEM workforce. The overrepresentation of Asians in STEM along with the model minority myth may contribute to these undergraduate students’ perceptions that scientists are often Asian. As important as it is to reject negative STEM stereotypes toward Black and Latino students it is also important to not endorse stereotypes that place unrealistic expectations on a group of people. Many Asian students have reported negative consequences, such psychological and emotional distress, due to the model minority myth and trying to fit into this image [30,39]. Therefore, dismantling stereotypes can benefit all students in STEM.

## Limitations

Consistent with much prior research using Draw-a-Scientist tasks [4,20], our sample was predominantly White given that this study was conducted at a predominantly White institution. Additionally, our sample had small numbers of Asian, Latino, and Black participants. Future research should consider including more diverse populations to gauge other ethnic minoritized groups’ perceptions of scientists. Additionally, collecting data from other universities which vary in terms of the ethnic representation of students served may also provide some insight on how students’ educational environments may influence their perceptions of scientists as well. Perhaps attending a university with more racial/ethnic diverse professors and staff or a minority serving institution where one’s peers are predominantly from groups historically excluded from STEM may promote more diverse representations of scientist avatars. Furthermore, our sample was mostly participants that identified as either male or female. There is a lack of work in this area that specifically examines trans or gender-nonconforming individuals’ perceptions of scientists. Therefore, future research should be more intentional about recruiting participants from these communities to better understand their perceptions and experiences in STEM. Lastly, this study included cross-sectional data thus future research should also consider collecting longitudinal data to see how these perceptions may unfold across the college period.

## Conclusion

Using a new method based on the Draw-a-Scientist task, this study collected college students’ perceptions of scientists and demonstrated that factors like color-blind attitudes and explicit STEM stereotypes were influential. Additionally, ingroup preferences also played an important role. Furthermore, using the new method, Design-a-Scientist Avatar, allowed participants to create an avatar of a scientist with an online application rather than the traditional paper measure. This app allowed us to assess the association between skin tone and ascribed race/ethnicity and demonstrated that there were similarities for White and Black scientist avatars. While still only a pilot validation, this study suggests this app has the potential to be a widely used measure for capturing stereotypes. Thus, through the use of the app, we may gain a more nuanced understanding of the STEM stereotypes that people may have and be better able to change such perceptions.

## References

[1] National Center for Science and Engineering Statistics. Women, Minorities, and Persons with Disabilities in Science and Engineering: 2021. Special Report NSF 21-321. 2021.

[2] Simon RM, Wagner A, Killion B. Gender and choosing a STEM major in college: Femininity, masculinity, chilly climate, and occupational values. J Res Sci Teach. 2016;54(3):299–323. doi: 10.1002/tea.21345

[3] Rainey K, Dancy M, Mickelson R, Stearns E, Moller S. Race and gender differences in how sense of belonging influences decisions to major in STEM. Int J STEM Educ. 2018;5(1):10. doi: 10.1186/s40594-018-0115-6 30631700 PMC6310405

[4] Miller DI, Nolla KM, Eagly AH, Uttal DH. The Development of Children’s Gender-Science Stereotypes: A Meta-analysis of 5 Decades of U.S. Draw-A-Scientist Studies. Child Dev. 2018;89(6):1943–55. doi: 10.1111/cdev.13039 29557555

[5] Neville HA, Awad GH, Brooks JE, Flores MP, Bluemel J. Color-blind racial ideology: theory, training, and measurement implications in psychology. Am Psychol. 2013;68(6):455–66. doi: 10.1037/a0033282 24016116

[6] Kellow JT, Jones BD. The Effects of Stereotypes on the Achievement Gap: Reexamining the Academic Performance of African American High School Students. Journal of Black Psychology. 2008;34(1):94–120. doi: 10.1177/0095798407310537

[7] Smeding A. Women in Science, Technology, Engineering, and Mathematics (STEM): An Investigation of Their Implicit Gender Stereotypes and Stereotypes’ Connectedness to Math Performance. Sex Roles. 2012;67(11–12):617–29. doi: 10.1007/s11199-012-0209-4

[8] Spencer SJ, Steele CM, Quinn DM. Stereotype threat and women’s math performance. Journal of Experimental Social Psychology. 1999;35(1):4–28.

[9] Schmader T. Gender identification moderates stereotype threat effects on women’s math performance. Journal of Experimental Social Psychology. 2002;38(2):194–201.

[10] Shapiro JR, Williams AM. The Role of Stereotype Threats in Undermining Girls’ and Women’s Performance and Interest in STEM Fields. Sex Roles. 2011;66(3–4):175–83. doi: 10.1007/s11199-011-0051-0

[11] Beasley MA, Fischer MJ. Why they leave: the impact of stereotype threat on the attrition of women and minorities from science, math and engineering majors. Soc Psychol Educ. 2012;15(4):427–48. doi: 10.1007/s11218-012-9185-3

[12] Neville HJ, Stevens C, Pakulak E, Bell TA, Fanning J, Klein S, et al. Family-based training program improves brain function, cognition, and behavior in lower socioeconomic status preschoolers. Proc Natl Acad Sci U S A. 2013;110(29):12138–43. doi: 10.1073/pnas.1304437110 23818591 PMC3718115

[13] Espinoza KJC, Rincón BE. Color-Evasive/Conscious? A Content Analysis of How Engineering Faculty Discuss Race and Racism in a U.S.-Based Equity-Focused STEM Professional Development Program. Education Sciences. 2023;13(3):233. doi: 10.3390/educsci13030233

[14] King GP, Russo-Tait T, Andrews TC. Evading Race: STEM Faculty Struggle to Acknowledge Racialized Classroom Events. CBE Life Sci Educ. 2023;22(1):ar14. doi: 10.1187/cbe.22-06-0104 36735542 PMC10074277

[15] Midgette AJ, Mulvey KL. Unpacking Young Adults’ Experiences of Race and Gender-Based Microaggressions. J Soc Pers Relat. 2021;38(4):1350–70. doi: 10.1177/0265407521988947 33927467 PMC8081288

[16] Thomas MD, Henley TB, Snell CM. The Draw a Scientist Test: A Different Population and a Somewhat Different Story. College Student Journal. 2006;40(1):140–8.

[17] Medina-Jerez W, Middleton KV, Orihuela-Rabaza W. Using the dast-c to explore colombian and bolivian students’ images of scientists. Int J of Sci and Math Educ. 2010;9(3):657–90. doi: 10.1007/s10763-010-9218-3

[18] Chambers DW. Stereotypic images of the scientist: The draw-a-scientist test. Science Education. 1983;67(2):255–65. doi: 10.1002/sce.3730670213

[19] Ferguson SL, Lezotte SM. Exploring the state of science stereotypes: Systematic review and meta-analysis of the Draw-A-Scientist Checklist. School Sci & Mathematics. 2020;120(1):55–65. doi: 10.1111/ssm.12382

[20] Walls L. A critical race theory analysis of the draw-a-scientist test: are they really that white?. Cult Stud of Sci Educ. 2022;17(1):141–68. doi: 10.1007/s11422-022-10107-6

[21] Arnett JJ. Emerging Adulthood: What Is It, and What Is It Good For?. Child Dev Perspectives. 2007;1(2):68–73. doi: 10.1111/j.1750-8606.2007.00016.x

[22] McLean KC, Koepf IM, Lilgendahl JP. Identity Development and Major Choice Among Underrepresented Students Interested in STEM Majors: A Longitudinal Qualitative Analysis. Emerging Adulthood. 2021;10(2):386–401. doi: 10.1177/21676968211015549

[23] Graham MJ, Frederick J, Byars-Winston A, Hunter A-B, Handelsman J. Science education. Increasing persistence of college students in STEM. Science. 2013;341(6153):1455–6. doi: 10.1126/science.1240487 24072909 PMC10167736

[24] strmic-pawl hephzibah v., Gonlin V, Garner S. Color in Context: Three Angles on Contemporary Colorism. Sociology of Race and Ethnicity. 2021;7(3):289–303. doi: 10.1177/23326492211012532

[25] Ma Y, Liu Y. Race and STEM Degree Attainment. Sociology Compass. 2015;9(7):609–18.

[26] von Luschan F. Beiträge zur völkerkunde der deutschen schutzgebiete.: D. Reimer (E. Vohsen). 1897.

[27] Jablonski NG. The Evolution of Human Skin and Skin Color. Annu Rev Anthropol. 2004;33(1):585–623. doi: 10.1146/annurev.anthro.33.070203.143955

[28] Rowley SJ, Kurtz-Costes B, Mistry R, Feagans L. Social Status as a Predictor of Race and Gender Stereotypes in Late Childhood and Early Adolescence. Social Development. 2007;16(1):150–68. doi: 10.1111/j.1467-9507.2007.00376.x

[29] Liben LS, Bigler RS. The developmental course of gender differentiation: conceptualizing, measuring, and evaluating constructs and pathways. Monogr Soc Res Child Dev. 2002;67(2):i–viii, 1–147; discussion 148-83. 12465575

[30] Yi V, Museus SD. Model minority myth. The Wiley Blackwell Encyclopedia of Race, Ethnicity, and Nationalism. 2015. p. 1–2.

[31] Kang C, Jo H, Han SW, Weis L. Complexifying Asian American student pathways to STEM majors: Differences by ethnic subgroups and college selectivity. Journal of Diversity in Higher Education. 2023;16(2):215–25. doi: 10.1037/dhe0000326

[32] Worthington RL, Navarro RL, Loewy M, Hart J. Color-blind racial attitudes, social dominance orientation, racial-ethnic group membership and college students’ perceptions of campus climate. Journal of Diversity in Higher Education. 2008;1(1):8–19. doi: 10.1037/1938-8926.1.1.8

[33] Neville HA, Poteat VP, Lewis JA, Spanierman LB. Changes in White college students’ color-blind racial ideology over 4 years: do diversity experiences make a difference?. J Couns Psychol. 2014;61(2):179–90. doi: 10.1037/a0035168 24635589

[34] Parks SJ, Yoo HC, Tran AGTT. Does color-blind racial ideology moderate the internalization of the model minority myth on race-related stress among Asian American college students?. Journal of Diversity in Higher Education. 2024;17(3):456–66. doi: 10.1037/dhe0000419

[35] Araujo-Dawson B. Understanding the Complexities of Skin Color, Perceptions of Race, and Discrimination Among Cubans, Dominicans, and Puerto Ricans. Hispanic Journal of Behavioral Sciences. 2015;37(2):243–56. doi: 10.1177/0739986314560850

[36] Sorge C, Newsom HE, Hagerty JJ. Fun is Not Enough: Attitudes of Hispanic Middle School Students toward Science and Scientists. Hispanic Journal of Behavioral Sciences. 2000;22(3):332–45. doi: 10.1177/0739986300223004

[37] Hernandez D, Rana S, Rao A, Usselman M. Dismantling Stereotypes About Latinos in STEM. Hispanic Journal of Behavioral Sciences. 2017;39(4):436–51. doi: 10.1177/0739986317731100

[38] National Science Board. The STEM Labor Force of Today: Scientists, Engineers and Skilled Technical Workers. Science and Engineering Indicators 2022 NSB-2021-2. Alexandria, VA.2021.

[39] McGee E. “Black Genius, Asian Fail”: The Detriment of Stereotype Lift and Stereotype Threat in High-Achieving Asian and Black STEM Students. AERA Open. 2018;4(4). doi: 10.1177/2332858418816658

